# The role of electrical stimulation in bone regeneration: mechanistic insights and therapeutic advances

**DOI:** 10.1186/s42234-025-00180-x

**Published:** 2025-08-08

**Authors:** Samira Farjaminejad, Aaron M. Dingle

**Affiliations:** 1 Department of Health Services Research and Management, School of Health and Psychological Sciences, University of London, London, WC1E 7HU UK; 2 Department of Surgery Division of Plastic Surgery, University of Wisconsin-Madison, Madison, WI USA

**Keywords:** Bone regeneration, Electrical stimulation, Osteogenesis, Direct current, Capacitive coupling, Pulsed Electromagnetic Field

## Abstract

Bone regeneration is a complex biological process that involves the coordinated action of osteoblasts, osteoclasts, and mesenchymal stem cells (MSCs). While bone possesses an intrinsic ability to heal, large defects, delayed unions, and non-unions require advanced therapeutic interventions. Electrical stimulation (ES) has emerged as a promising strategy to enhance bone healing by modulating cellular activity, promoting osteogenic differentiation, and accelerating vascularization. This review explores the mechanistic role of bioelectrical cues in bone regeneration, emphasizing the influence of voltage-gated ion channels, particularly voltage-gated calcium channels (VGCCs), in transducing electrical signals into biochemical responses. Various types of ES modalities, including direct current (DC), capacitive coupling (CC), Pulsed Electromagnetic Field (PEMF), and piezoelectric stimulation, are evaluated for their effectiveness in clinical and preclinical applications. Additionally, the synergistic potential of ES when combined with biomaterials, stem cells, and growth factors is discussed. Despite promising results, challenges remain in translating preclinical findings to clinical applications, with key hurdles including standardization of treatment protocols, variability in patient responses, and regulatory constraints. Large-animal models have provided insights into the efficacy of ES-based therapies, but limitations in field penetration and treatment reproducibility hinder widespread adoption. Future advancements in bioelectronic medicine, smart scaffolds, and artificial intelligence (AI)-driven personalized therapies hold potential to optimize ES-based bone regeneration. Addressing current limitations through interdisciplinary research will be critical in establishing ES as a mainstream therapeutic approach in orthopedic and maxillofacial regenerative medicine.

**Graphical Abstract:**

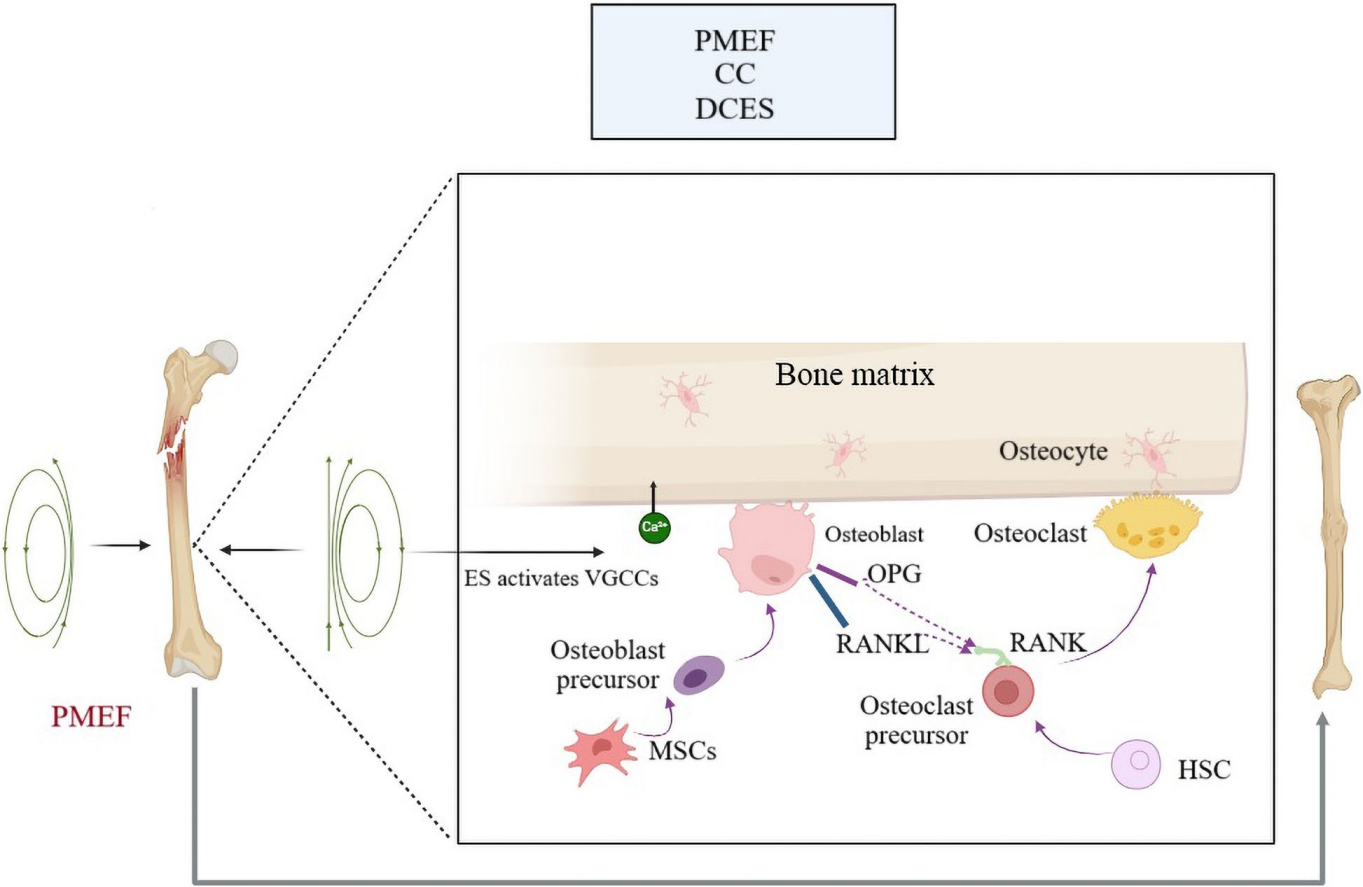

**Supplementary Information:**

The online version contains supplementary material available at 10.1186/s42234-025-00180-x.

## Introduction

Bone tissue has a natural ability to regenerate. In critical-sized defects, delayed unions, and non-union fractures, the natural healing process is inadequate, requiring medical intervention. Traditional approaches, including bone grafting, tissue engineering (TE), and ES, have been used to enhance healing, but each has limitations (Al-Himdani et al. 2017; Anand et al. 2023). Autografts, allografts, and synthetic bone substitutes have been extensively used in orthopedic, maxillofacial, and spinal surgeries. However, they often suffer from limitations such as donor site morbidity, immune rejection, and poor integration (Farjaminejad et al. 2024a; Sun et al. 2023; Herculano et al. 2009). To address these challenges, BTE has gained significant attention, incorporating scaffold-guided regeneration, growth factor delivery, and stem cell-based therapies (Farjaminejad et al. 2024b; Gillman And Jayasuriya 2021). Additionally, ES has emerged as a promising strategy to enhance cellular activity, matrix mineralization, and angiogenesis, improving overall bone healing outcomes (Sun et al. 2023; Herculano et al. 2009).

Despite these advances, major challenges remain, including insufficient vascularization, mechanical instability at fracture sites, and impaired osteogenic cell recruitment and differentiation all of which negatively impact bone regeneration (Al-Himdani et al. 2017; Herculano et al. 2009; Ogay et al. 2020). Furthermore, suboptimal biomaterial integration due to mismatches in biodegradation and immune rejection limits the effectiveness of synthetic substitutes (Sun et al. 2023; Gu et al. 2013). While bone grafting, stem cell therapy, and biomaterial scaffolds have significantly improved outcomes, alternative approaches, such as bioelectrical stimulation, are gaining attention as promising adjunct therapies for complex fractures and non-union cases (Anand et al. 2023; Krech et al. 2020).

This review provides an analysis of ES in bone regeneration, exploring its biological mechanisms, clinical applications, and challenges in treating critical-sized defects and non-unions. It examines the effects of ES on osteogenesis, angiogenesis, and inflammation, as well as various ES techniques and their clinical relevance. Additionally, the review discusses integrative approaches that combine ES with biomaterials, stem cells, and mechanical loading. Finally, it addresses key challenges, including treatment standardization and clinical translation, highlighting the need for further research to optimize ES-based therapies in regenerative medicine and orthopedics.

## Bone regeneration mechanisms

### Bone healing process: inflammation, repair, and remodeling

**Fig. 1:**
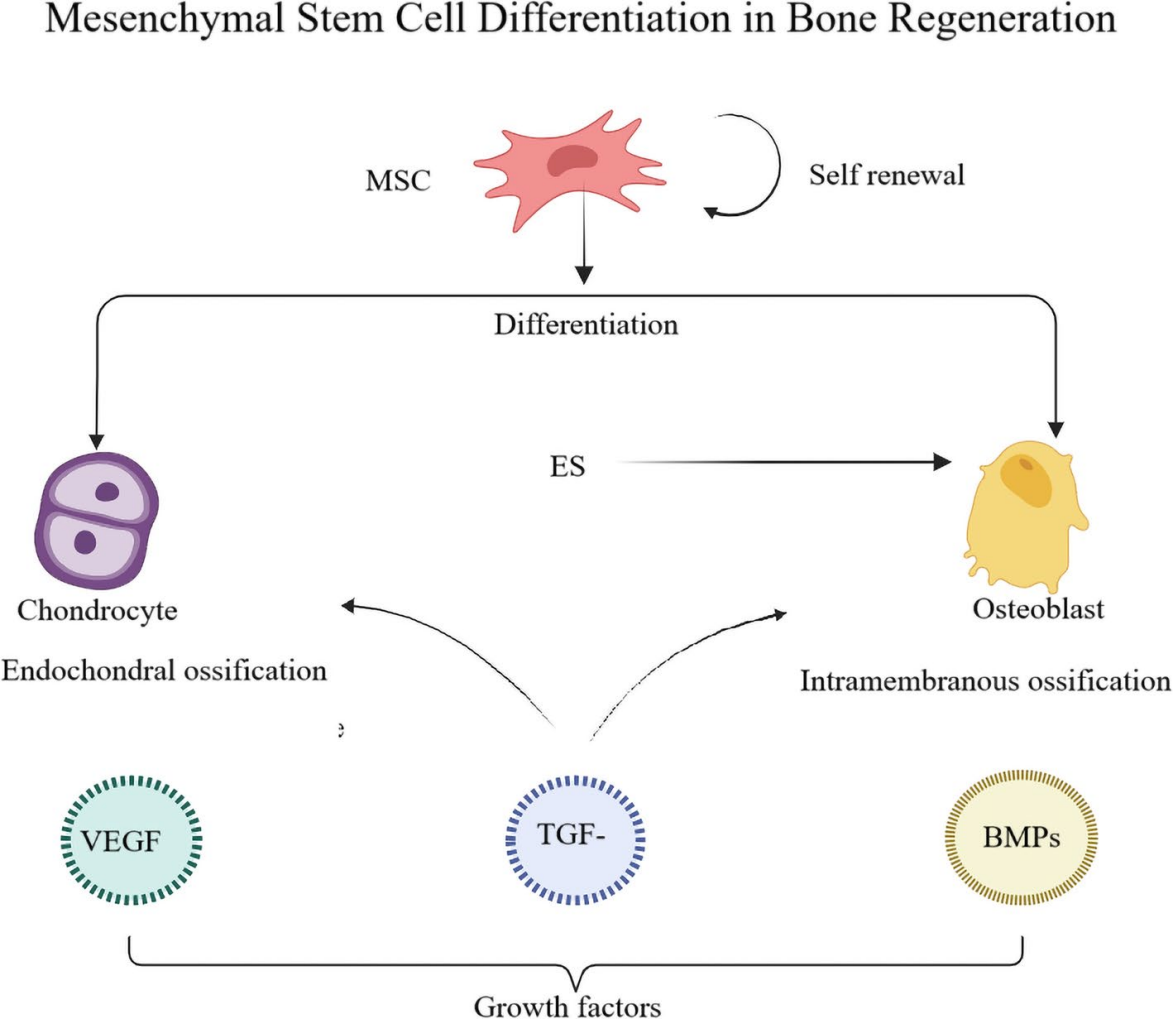
Schematic representation of MSC differentiation in bone regeneration. MSCs can either self-renew or differentiate into chondrocytes and osteoblasts, contributing to endochondral and intramembranous ossification, respectively. Growth factors such as VEGF, TGF-β, and BMPs regulate these pathways. ES enhances osteogenic signaling and supports bone matrix formation

Bone regeneration is a complex, tightly regulated process that restores skeletal integrity after injury. It occurs in three overlapping phases: inflammation, repair, and remodeling, each involving distinct cellular and molecular interactions vital for restoring bone structure and function (Loi et al. 2016; Elhawary et al. 2021; Newman et al. 2021; Diomede et al. 2020). Osteoblasts, derived from MSCs, produce type I collagen, osteocalcin, and alkaline phosphatase to form the mineralization scaffold. Their differentiation is regulated by Runx2 and Osterix, activated by TGF-β and BMPs (Ottewell 2016; Chen et al. 2018; Wang et al. 2013). Osteoclasts resorb mineralized bone and release TGF-β and IGF-1, indirectly affecting osteoblasts. Bone formation and resorption are balanced through the RANKL/OPG pathway (Abbondati 2022). MSCs, precursors to osteoblasts and chondrocytes, respond to growth factors, driving bone repair and differentiation (Fig. 1) (Iaquinta et al. 2021). VEGF, BMPs, and TGF-β also regulate angiogenesis, osteogenesis, and matrix deposition, further influencing remodeling (Ottewell 2016). Table 1 summarizes these functions.

**Table 1:** Key growth factors and their functions in bone healing

Growth Factor	Primary Function	Phase of Bone Healing	Ref
VEGF	Stimulates angiogenesis Enhances vascular supply	Inflammation Repair	(Loi et al. 2016; Newman et al. 2021)
BMPs	Induces osteoblast differentiation and bone formation	Repair	(Elhawary et al. 2021; Newman et al. 2021)
TGF-β	Regulates MSC proliferation Promotes collagen deposition	Repair Remodeling	(Loi et al. 2016; Ottewell 2016 )
IL-6	Pro-inflammatory cytokine recruits MSCs and immune cells	Inflammation	(Newman et al. 2021; Wang et al. 2013)
TNF-α	Initiates inflammation Regulates osteoclast activation	Inflammation	(Loi et al. 2016; Chen et al. 2018 )
IL-10	Suppresses inflammation, promotes healing	Repair	(Newman et al. 2021; Ottewell 2016)

## Bone physiology and electrical cues

Bone is a tissue with mechanical and electrical properties vital to regeneration. It comprises an organic matrix (mainly type I collagen) and an inorganic phase (hydroxyapatite), which provide strength and support bioelectrical signals that influence cell communication, differentiation, and remodeling (Sun et al. 2024; Strangis et al. 2024). Bone’s piezoelectricity, first described by Fukada and Yasuda, results from stress-induced charge displacement in the collagen-HA matrix. This endogenous bioelectricity regulates the activity of osteoblasts, osteoclasts, and MSCs, influencing bone formation, resorption, and repair (Zaszczy´nska et al. 2024; Farjaminejad et al. 2025).

A study by Strangis, G., et al. showed that piezoelectric signals influence bone regeneration through calcium signaling pathways, including the activation of VGCCs (Strangis et al. 2024). The piezo-electric effect in bone is primarily attributed to collagen fibrils, which undergo shear deformation and charge redistribution when subjected to external forces. This charge displacement results in the generation of localized electrical fields, which influence bone remodeling and fracture healing (Strangis et al. 2024; Zaszczy´nska et al. 2024). Furthermore, HA, as the mineral component of bone, interacts with the collagen matrix to modulate electrical conductivity and ion exchange, further enhancing cell responses and tissue regeneration (ElyaderAni et al. 2022).

## Mechanisms of ES in bone regeneration

**Fig. 2:**
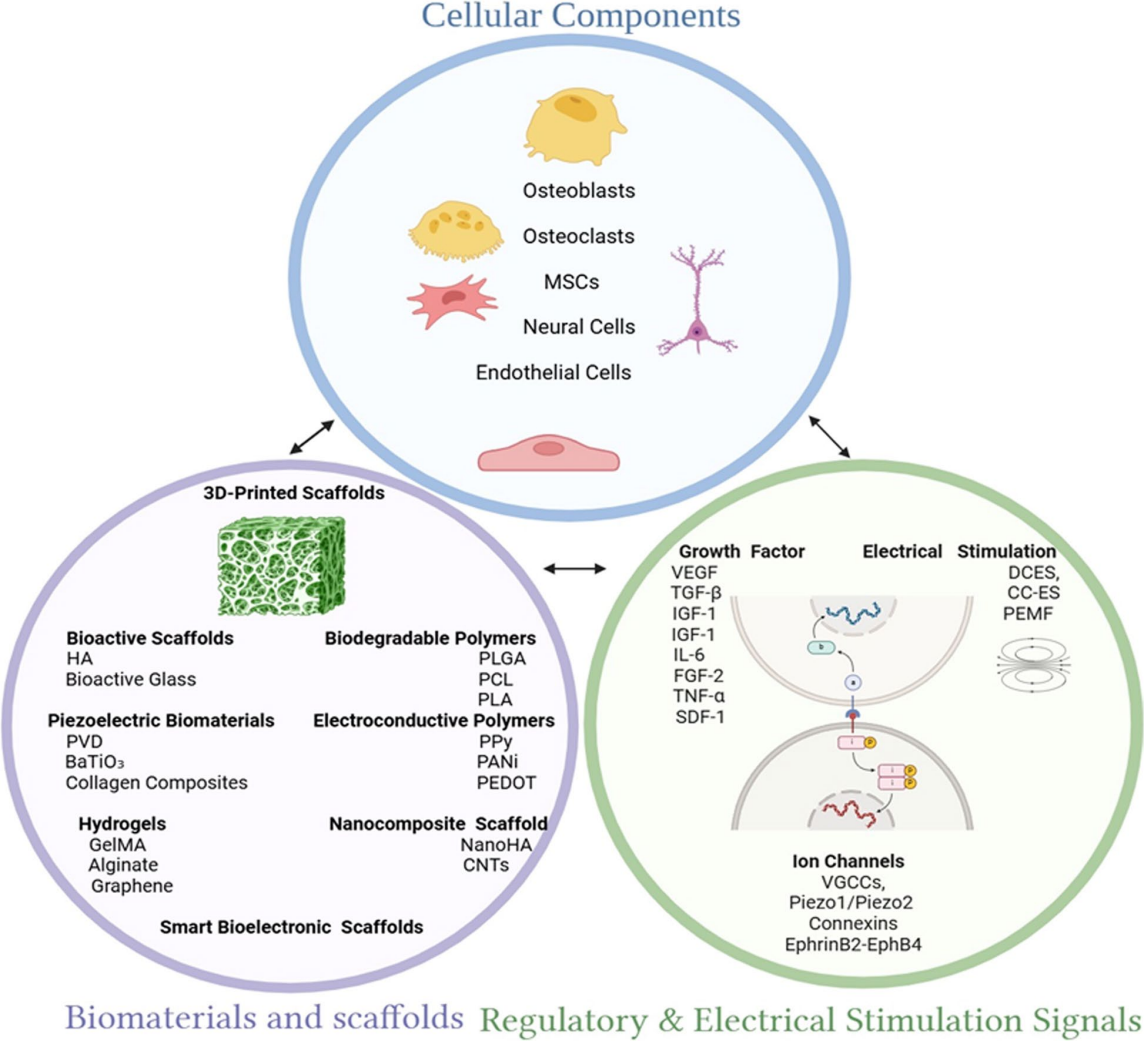
**a** Cellular components (blue circle): This section encompasses key cell types involved in bone regeneration and responses to electrical cues, including osteoblasts (bone-forming cells), osteoclasts (bone-resorbing cells), MSCs (multipotent precursors), neural cells (which interact with bioelectric signals), and endothelial cells (critical for angiogenesis). These cells coordinate remodeling and regeneration in response to both mechanical and electrical stimulation. **b** Biomaterials and Scaffolds (purple circle): Various materials are incorporated into scaffolds to provide structural and functional support. These include: bioactive scaffolds (e.g., HA, bioactive glass) to promote mineralization; biodegradable polymers (e.g., PLGA, PCL, PLA) for controlled degradation; piezoelectric biomaterials (e.g., PVD, BaTiO₃, collagen composites) that generate electric charges under mechanical loading; electroconductive polymers (e.g., PPy, PANi, PEDOT) to facilitate electrical signal transmission; nanocomposite scaffolds (e.g., NanoHA, CNTs) to enhance mechanical strength and osteoinductivity; and hydrogels (e.g., GelMA, alginate, graphene) to support cell encapsulation and nutrient diffusion. These materials form the foundation for smart, bioelectronic scaffolds used in ES-enhanced bone regeneration. **c** Regulatory and ES Signals (green circle): This area outlines the external and internal regulatory cues affecting bone healing. It includes growth factors (e.g., VEGF, TGF-β, IGF-1, IL-6, FGF-2, TNF-α, SDF-1) that stimulate angiogenesis, differentiation, and matrix formation; ES modalities (DCES, CC-ES, PEMF) that modulate cell behavior through ionic movement and mechanotransduction; and ion channels (e.g., VGCCs, Piezo1/Piezo2, connexins, EphrinB2-EphB4) that transduce electrical cues into intracellular responses, regulate calcium signaling, and influence osteogenesis and remodeling

ES promotes osteogenesis and angiogenesis by modulating cellular responses and intracellular signaling. Studies show that electrical fields influence the differentiation and activity of osteoblasts, osteoclasts, and MSCs (Sun et al. 2023; Gu et al. 2013; Sahm et al. 2022). The application of ES enhances the expression of BMPs, VEGF, and Wnt/TGF-β-catenin signaling, all of which contribute to bone healing (Sahm et al. 2022). Figure 2 illustrates the key cellular components, biomaterials, and electrical stimulation strategies in BTE.

VGCCs are key in converting electrical signals into biochemical responses that support mineralization and bone matrix deposition (Nicksic et al. 2022a; Verma et al. 2022). L-type VGCCs regulate calcium (Ca2 +) influx in MSCs and osteoblasts, activating BMP and Wnt/β-catenin pathways to enhance osteogenesis (Table 2) (Ahamad And Singh 2021). Other subtypes, like P/Q-type (Cav2.1) and N-type (Cav2.2), promote calcium influx in osteoblasts, influencing proliferation and differentiation, and regulate calcium oscillations in osteoclasts to control resorption. T-type VGCCs (Cav3. x) aid osteogenic differentiation and early osteoclast fusion, while R-type (Cav2.3) supports cytoskeletal remodeling, affecting osteoblast adhesion and osteoclast polarization (Ahamad And Singh 2021).

**Table 2:** VGCCs and their roles in bone remodeling

VGCC Subtype	Osteoblast Role	Osteoclast Role	Ref
P/Q-Type (Cav2.1)	Regulates proliferation and differentiation	Modulates vesicle trafficking and resorption	(Ahamad And Singh 2021)
N-Type (Cav2.2)	Supports calcium-dependent signaling	Regulates intracellular calcium oscillations	(Ahamad And Singh 2021)
T-Type (Cav3.x)	Enhances early-stage osteoblast differentiation	Essential for osteoclastogenesis and fusion	(Ahamad And Singh 2021)
R-Type (Cav2.3)	Facilitates adhesion and matrix deposition	Involved in cytoskeletal rearrangement and resorption	(Ahamad And Singh 2021)
IP3R1/IP3R2	Not well-characterized in osteoblasts	Regulates differentiation and calcium-dependent resorption	(Ahamad And Singh 2021)

The piezoelectric properties of bone enable it to generate electric fields in response to mechanical loading, directly influencing the behavior of osteoblasts, osteoclasts, and MSCs, and interacting synergistically with biochemical factors to optimize bone repair and remodeling (Luo et al. 2024). Collagen, as a piezoelectric polymer, generates biological charges by rearranging dipole alignment under mechanical stress, producing surface charges that promote cell adhesion and mineralization (Zaszczy´nska et al. 2024). HA, the mineral component of bone, exhibits semiconducting behavior, facilitating bioelectrical signal transmission and regulating ion exchange—particularly calcium and phosphate transport—essential for mineral deposition and bone growth (Kahil et al. 2021).

Electrical fields, combined with mechanical loading and biochemical cues, create a synergistic effect in bone repair (Verma et al. 2022; Nicksic et al. 2022a). Targeting VGCCs through bioelectrical stimulation or pharmacological agents offers a promising strategy for enhancing bone regeneration in regenerative medicine (Ahamad And Singh 2021). ES modulates inflammatory responses, creating a microenvironment that supports healing and limits prolonged damage (Niu et al. 2021). It also accelerates mineral deposition and bone matrix remodeling, enhancing the mechanical strength and structural integrity of regenerated bone (Yao et al. 2025). Leveraging bone bioelectrical properties, researchers are developing self-powered scaffolds and bioelectronic devices to improve scaffold integration, modulate cellular responses, and accelerate bone regeneration in TE applications (Zhang et al. 2023).

## Types of ES for bone regeneration

**Fig. 3:**
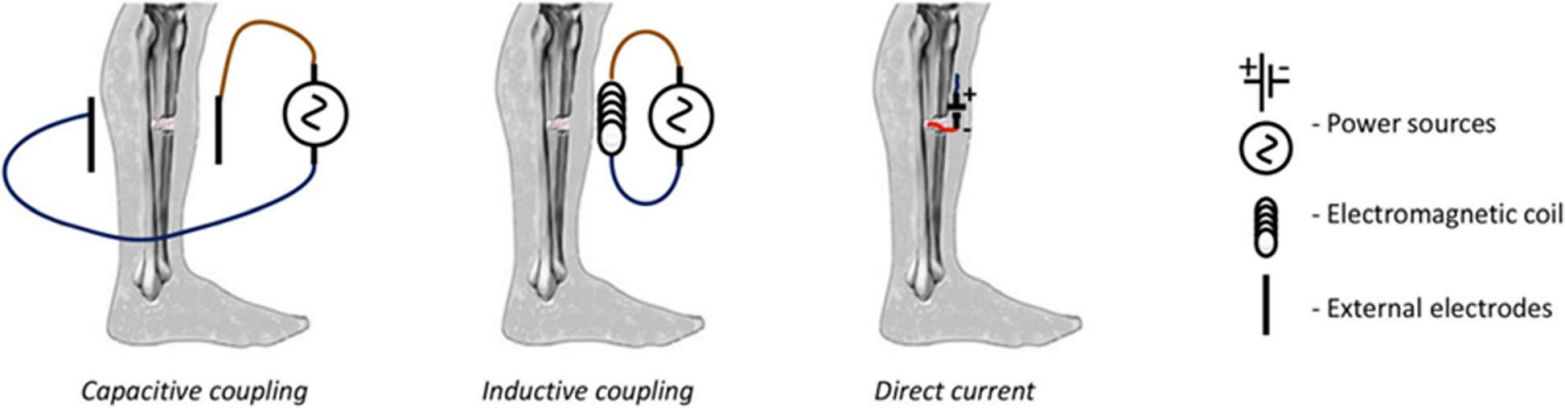
The figure illustrates three main ES methods for bone regeneration: (1) CC, applying alternating electrical fields via external electrodes; (2) PEMF, using an external coil to generate electromagnetic fields that induce currents in bone; and (3) DCES, delivering continuous low-intensity DC through implanted electrodes at the fracture site. Each method promotes osteogenesis by modulating cellular responses, enhancing matrix mineralization, and supporting osseointegration. Adapted from (Zhang et al. 2023)

Advancements in bioelectronics, TE, and personalized medicine help overcome limitations in bone regeneration therapies. With clinical trials and technological progress, electrical stimulation could become an effective adjunct for complex fractures and orthopedic reconstruction (Nicksic et al. 2022a; Verma et al. 2022; Nicksic et al. 2022b). ES has been explored as a non-invasive or minimally invasive adjunct therapy to enhance fracture healing and osseointegration. Clinical applications of ES, including pulsed electromagnetic fields, capacitive coupling, and direct current stimulation (Fig. 3), (Table 3), have demonstrated significant potential in fracture healing, non-union repair, and orthopedic implant integration, providing a non-invasive or minimally invasive adjunct therapy for enhanced bone regeneration (Anand et al. 2023; Gu et al. 2013; Leppik et al. 2020).

**Table 3:** Types of ES in bone regeneration

ES Type	Principles & Mechanism	Effects on Bone Cells	Clinical Applications	Challenges & Limitations	Ref
DC	Continuous low-intensity DC applied via implantable electrodes; mimics natural bone bioelectric fields	Enhances osteoblast activity Increases calcium influx Activates BMP, TGF-β, Wnt/β-catenin pathways	Used in spinal fusion and non-union fractures, it accelerates mineralization but requires surgical implantation	Invasive, risk of infection, electrode failure; patient compliance issues	(Nicksic et al. 2022a; Verma et al. 2022; Nicksic et al. 2022b)
CC	Alternating electric field generated by external electrodes; non-invasive, stimulates osteoblast proliferation	Stimulates osteogenic differentiation Increases BMP2 and VEGF expression Enhances MSC recruitment	Effective in osteoporosis-related fractures, spinal fusion; limited penetration depth affects deep bone healing	Limited penetration, inconsistent response among patients, long treatment duration required	(Nicksic et al. 2022a; Verma et al. 2022; Nicksic et al. 2022b)
PEMF	EMFs induce weak electrical currents within bone, promoting osteogenesis and angiogenesis	Promotes osteoblast differentiation, chondrogenesis, and vascularization; activates Wnt/β-catenin and TGF-β signaling	FDA-approved for long bone fractures, spinal fusion; shows success in non-union treatment but inconsistent outcomes in large defects	Variability in treatment parameters, inconsistent success in large bone defects, lack of standardization	(Nicksic et al. 2022a; Verma et al. 2022; Nicksic et al. 2022b)

### Direct Current Electrical Stimulation (DCES)

DCES involves the continuous application of low-intensity DC to the bone surface through implantable electrodes at the fracture site. This method generates a steady electrical field, stimulating osteogenesis, enhancing osteoblastic activity, promoting bone cell proliferation, and accelerating fracture healing in both animal models and clinical trials (Nicksic et al. 2022a; Verma et al. 2022). Clinical studies suggest that DCES is particularly beneficial for non-union fractures and spinal fusion surgeries, as it promotes bone mineralization and supports long-term bone regeneration (Abbondati 2022). However, its invasive nature, along with risks such as infection, device failure, and high costs, has limited its widespread clinical use (Verma et al. 2022).

### Capacitive Coupling (CC) electrical stimulation for osseointegration

CC stimulation uses skin-placed electrodes to generate an alternating electrical field near the fracture site, modulating cellular activity. This non-invasive method has shown promise in enhancing osteogenic differentiation and improving implant osseointegration, especially in spinal fusion cases (Nicksic et al. 2022a).

Clinical evidence supports CC therapy for osteoporotic fractures and implant integration; however, limited field penetration and patient compliance hinder its broader adoption (Nicksic et al. 2022b). Although preclinical studies in small animals have shown positive effects of ES, clinical translation to large-animal and human models has faced challenges. Weaker electric fields at the fracture site in humans may explain the limited clinical success of non-invasive CC therapies (Verma et al. 2022). A computational modeling study comparing sheep and human cadaver models showed that current non-invasive Electronic Bone Growth Stimulator (EBGS) devices fail to deliver adequate electric fields to deep bone fractures. To overcome this, researchers have proposed the Injectrode an injectable electrode system to enhance field penetration and improve the clinical effectiveness of ES therapies (Verma et al. 2022).

### Pulsed Electromagnetic Field (PEMF) therapy fracture healing

PEMF, a form of inductive coupling, was first approved by the FDA in 1979 to treat non-union of bone including failed fusions and congenital pseudoarthrosis. This non-invasive therapy generates electromagnetic fields (EMFs) to stimulate osteoblastic activity and enhance vascularization, making it particularly useful in cases of delayed union and non-union fractures (Nicksic et al. 2022a; Verma et al. 2022). Clinical trials have demonstrated that PEMF reduces the time required for fracture healing, particularly in long bone fractures and osteotomies. However, variations in treatment parameters and device specifications have contributed to inconsistent clinical outcomes, warranting further optimization (Nicksic et al. 2022a; Verma et al. 2022). One study suggested that PEMF is more effective in spinal fusion than in long bone fractures, highlighting the need for optimized stimulation parameters and patient selection to improve clinical efficacy (Javeed et al. 2021).

## Synergistic effects of ES in bone regeneration

The synergistic effects of ES with other bone regenerative strategies have been extensively studied. ES, when combined with growth factors such as bone morphogenetic proteins (BMPs) and VEGF, enhances cellular responses, leading to improved bone healing efficiency (Sun et al. 2023; Ogay et al. 2020). Additionally, scaffold-based approaches that incorporate bioactive materials have demonstrated improved osteoconductivity and osteoinductivity when coupled with ES, facilitating better integration and bone tissue formation (Herculano et al. 2009). Recent advancements in three-dimensional (3D)-printed with embedded ES components further enable personalized regenerative therapies, offering tailored solutions for complex bone defects (Sun et al. 2023; Ogay et al. 2020). Beyond biochemical signaling, the interaction between ES and mechanical loading has gained attention, as their combined effects more closely mimic the natural biomechanical environment of bone tissue, further enhancing remodeling and regeneration (Nicksic et al. 2022a; Verma et al. 2022). Despite promising results, challenges such as optimizing electrical parameters, ensuring long-term scaffold stability, and patient-specific variability remain key areas of research.

### Scaffold-based bone regeneration in clinical applications

Scaffold-guided bone regeneration has been widely applied in orthopedic, maxillofacial, and spinal surgeries to facilitate bone healing. Recent clinical trials evaluating 3D-printed polymeric and ceramic scaffolds have demonstrated improved osteoconductivity and osteoinductivity, particularly when combined with bioactive factors such as BMPs and MSCs (Laubach et al. 2023). However, challenges such as vascularization, mechanical stability, and controlled biodegradation must be addressed to ensure long-term clinical success (Garot et al. 2021).

Among scaffold materials, calcium phosphate-based ceramics, such as HA, beta-tricalcium phosphate (β-TCP), and biphasic calcium phosphate have shown strong clinical relevance due to their biocompatibility and osteoconductive properties (Venkataiah et al. 2021). While many studies have focused on combining these ceramics with bone marrow-derived MSCs, others have sought to improve regenerative outcomes through additional modifications. Dilogo et al. incorporated BMP2 into cell-scaffold constructs to accelerate bone formation (Venkataiah et al. 2021; Dilogo et al. 2019). Enhanced osteogenesis has been reported using collagen-based scaffolds integrated with BMMSCs enriched with BMP2. Another notable approach by Baba et al. involved the development of a polylactic acid scaffold combined with BMMSCs and platelet-rich plasma (PRP), further optimized by adding human thrombin dissolved in calcium chloride 10% to improve regenerative potential (Venkataiah et al. 2021; Baba 2016). These approaches highlight continuous advancements in scaffold-based therapies. However, despite promising results, the absence of bioelectrical cues in traditional scaffold-based therapies presents a limitation in mimicking natural bone healing mechanisms.

### Scaffolds with ES for enhanced bone regeneration

#### Piezoelectric scaffolds in BTE

Piezoelectric scaffolds have emerged as a promising strategy to overcome the limitations of conventional scaffold-based therapies. These scaffolds are designed to convert mechanical stress into localized electrical signals, thereby recreating the bioelectrical environment essential for bone healing (Verma et al. 2022; Nicksic et al. 2022b). By mimicking the natural piezoelectric behavior of bone, these materials promote osteoblast proliferation, osteogenic differentiation, and mineralization in both in vitro and in vivo models (Venkataiah et al. 2021).

**Fig. 4:**
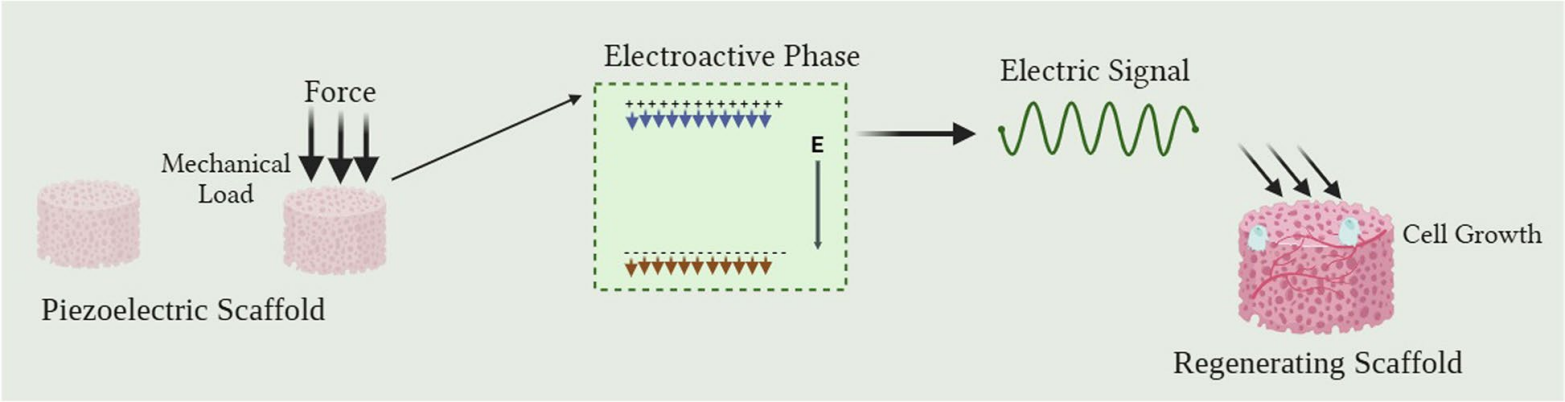
Schematic illustration of the piezoelectric effect in a scaffold-based BTE approach. Mechanical loading on the piezoelectric scaffold causes dipole realignment within the electroactive phase, leading to the generation of an internal electric field (E) and the emission of localized electrical signals. These signals act as bioelectrical cues that stimulate cell growth and tissue regeneration. The figure illustrates how mechanical energy can be transduced into electrical stimulation within the scaffold, thereby mimicking the natural bioelectrical environment of bone tissue. This figure was created using the BioRender.com platform

The piezoelectric effect in bone primarily originates from collagen fibrils, which generate electrical charges upon mechanical deformation, influencing cell adhesion, migration, and differentiation (Fig. 4) (Rajabi et al. 2015). However, scaffolds alone often face challenges such as insufficient vascularization, poor mechanical properties, and limited cell recruitment. To address these limitations, the integration of ES with scaffold-based therapies has gained increasing attention (Das et al. 2020). ES enhances osteogenesis, accelerates mineralization, and improves scaffold integration by replicating the natural bioelectrical signals present in bone tissue (Das et. al. 2020).

Recent advancements leverage 3D-printing technologies and piezoelectric biomaterials to develop scaffolds that not only provide mechanical support but also actively stimulate bone regeneration. Common piezoelectric ceramics used in this context include barium titanate, lithium sodium potassium niobate (LNKN), lithium niobate (LN), and potassium sodium niobate (KNN) (Li et al. 2023; Jianqing et al. 1997). Barium titanate (BaTiO_3_) nanoparticles have been widely utilized as a piezoelectric material due to their attractive properties, including a high piezoelectric coefficient and excellent biocompatibility (Li et al. 2023; Yu et al. 2012). In addition, various piezoelectric materials, including poly (L-lactic acid) (PLLA), HA-based composites, BaTiO_3_, and collagen-based composites, have been developed for scaffold fabrication. These materials generate endogenous electrical signals upon mechanical loading, mimicking the natural bioelectric environment of bone. They self-stimulate bone healing without the need for external power sources, offering a potential breakthrough in scaffold-based therapies (Das et al. 2020). A study by Silva C. A. et al. showed that dynamically stimulated piezoelectric scaffolds can increase osteoblast proliferation by approximately 20%, highlighting their potential in enhancing cell adhesion, proliferation, and matrix mineralization in BTE (Silva et al. 2022).

### Electroactive scaffolds in BTE

Electroactive scaffolds are biomaterials that conduct or respond to electrical signals, promoting cell proliferation, differentiation, and ECM deposition. ES applied via these scaffolds activates VGCCs, increases intracellular calcium, and upregulates osteogenic markers like Runx2, ALP, and osteocalcin, enhancing bone formation. It also stimulates pro-angiogenic factors such as VEGF, supporting vascularization and nutrient supply for sustained regeneration (Das et al. 2020).

Conductive polymers such as polypyrrole (PPy), polyaniline (PANi), and poly (3,4- ethylenedioxythiophene) (PEDOT) have been widely studied for their conductivity and biocompatibility, supporting osteogenesis and BTE applications (Das et al. 2020). Table 4 summarizes their electrical properties and bone healing effects. Combining ES with conductive polymer scaffolds amplifies local electric fields, boosting osteogenic activity by promoting mineral nucleation, protein adsorption, Ca2 + transport, and mitochondrial function (Huang et al. 2019; Jing et al. 2019; Zhu et al. 2017).

**Table 4:** Types of electroactive scaffolds

Scaffold Material	Electrical Properties	Effects on Bone Healing	Ref
PLLA/PPy	Conductivity: 0.094 ± 0.026 S/cm	Enhanced osteogenesis, mineralization	(Das et al. 2020)
PEDOT-PU	Resistance: 17 kΏ	Improved cell viability, osteoblast differentiation	(Dixon and Gomil Lion 2023)
CNT/HAp	Conductivity: 0.88 S/m	Promotes biomineralization, protein absorption	(Das et al. 2020)
PCL/MWCNT	Conductivity: 12.7 S/m	Increased osteogenic gene expression, ECM deposition	(Das et al. 2020)
PVDF-TrFE	Piezoelectric coefficient: ~ 20 pC/N	Generates electric fields under mechanical loading, supports cell adhesion	(Das et al. 2020; Silva et al. 2022)
PVDF	Electroactive β-phase enhancement	Enhances osteoblast proliferation by ~ 20%, mimics bone’s mechano-electric microenvironment	(Silva et al. 2022)
BaTiO₃	High dielectric constant, piezoelectric	Stimulates osteogenic differentiation, enhances calcium ion influx	(Das et al. 2020)

Several studies have confirmed the regenerative potential of piezoelectric polymer scaffolds. PVDF scaffolds alone could not induce apatite nucleation in simulated body fluid, even with ES; however, incorporating HA enabled successful mineralization, highlighting the benefits of composite systems (Malherbi et al. 2022; Bretcanu et al. 2009). These findings confirm ES’s beneficial role in bone regeneration, and its influence on cell movement (Murillo et al. 2017; Martino et al. 2018; Mycielska And Djamgoz 2004). Negatively charged PVDF scaffolds also promoted osteogenic differentiation in human adipose-derived stromal cells, enhancing cell adhesion and matrix mineralization (Ribeiro et al. 2018). In vivo studies in Wistar rats further demonstrated that poled PVDF-based scaffold implants resulted in greater formation of bone marrow and trabecular bone than controls (without any implant) over four weeks, whereby non-poled PVDF-based scaffolds resulted in no bone growth (Ribeiro et. al. 2017).

EMFs provide an additional stimulus to enhance the performance of piezoelectric scaffolds. While some studies report potential physiological disruptions, low-frequency EMFs have shown benefits for bone regeneration (Zaszczy´nska et al. 2024; Maziarz et. al. 2016; Saliev et al. 2014). One study found that dental pulp stem cells cultured on piezoelectric scaffolds showed enhanced attachment, viability, and protein adsorption under EMF exposure (Bar et al. 2023; Mirzaei et al. 2019).

## Preclinical and clinical applications

### In vivo studies and animal models for evaluating ES

Preclinical studies on electrical bone growth stimulators provide valuable insights despite variations in evaluation criteria, device types, and stimulation protocols (Verma et al. 2022). In-vitro studies show that DCES, CC, and PEMF positively influence osteogenesis related markers across cell types. These effects on cell proliferation and differentiation have been confirmed in small animal models (rodents), with histomorphological evidence of enhanced bone healing (Nicksic et al. 2022b). However, rodents’ small size limits translating to humans, as electric field distribution and mechanical loading differ significantly.

Large animal models (ovine, porcine, canine) are more clinically relevant due to skeletal size, remodeling rates, and loading conditions closer to humans (Verma et al. 2022; Nerubay et al. 1986; Srivastava et al. 1982). Studies in sheep show ES improves fracture healing and implant integration (Verma et al. 2022). Porcine models help assess load-bearing defects and scaffold integration under stimulation (Nerubay et al. 1986).

Early DCES studies in canine tibial osteotomy models showed significant histological improvement (Paterson et al. 1977). CC studies in rat epiphyseal plate cells reported enhanced bone elongation above 500 V/cm (Paterson et al. 1977). However, large-animal PEMF studies show mixed results; some sheep tibial osteotomy models report no radiographic or histological improvements (HT, AIMIHSSACMMHL 1985). Table 5 summarizes key findings, parameters, and outcomes. For the complete dataset, including additional studies, refer to Supplementary Table 5. Delivering adequate electric fields to deep bone remains challenging in large animals due to bone thickness and density. These models, while translationally valuable, are expensive, raise ethical concerns, and face logistical hurdles (Verma et al. 2022; Nicksic et al. 2022b).

**Table 5:** Summary of experimental studies on electrical stimulation for bone healing across different models

Year	Stimulation Type & Parameters	Model type	Osseous Injury	Duration	Outcome	Ref
1977	DCES; Stainless-steel cathode; 20 µA	Dog	Tibial gap osteotomy	58 days	Histological analysis: Enhanced fibrous tissue formation and endochondral ossification (*p* = 0.042); visual assessment: Significantly improved bone healing score (*p* < 0.01)	(Paterson et al. 1977)
1986	DCES; stainless-steel cathodes, 20 µA	Porcine 1- month-old	Lumbar fusion	0–56 days	Improved healing: Assessed by radiographic fusion score (*p* =.037) Increased osteoblastic activity: Confirmed by histomorphology scoring (*p* <.01)	(Nerubay et al. 1986)
1998	PEMF; 15 Hz, 4.5 ms	Rat osteoclasts		Once 18 h	1.8 mT stimulation: Led to a twofold increase in bone resorption (*p* <.009)	(Shankar et al. 1998)
2009	PEMF; 0.13 mT, 7.5 Hz, Efield: 2 mV/cm, 300 µs quasi-rectangular pulses	Human MSCs		2 h/day 10 days	Day 7: Control had 84% more cells (*p* <.05); ALP increased by 82% in PEMF (*p* <.01); day 10: PEMF group had 62% more cells (*p* <.05); Control showed 123% cell increase	(Tsai et al. 2009)
2010	CC; 10 V, 16 Hz	Rat, Adult Female	Osteoporosis	2 h/day 60 days	Bone mineral content: Increased, confirmed by X-ray diffraction (*p* <.01); bone mineral density: Significantly higher (*p* <.001)	(Lin and Lin 2011)
2014	PEMF; 1500 μA, 12.5 Hz	Sheep 62–70 kg 2 years old	Tibial osteotomy	12 h/a day	Callus maturation: Increased, confirmed by histology (*p* <.0001); radiodensity analysis: active stimulation group showed increased opacity (*p* <.0043)	(Muttini et al. 2014)
2015	PEMF; 1.5 ± 0.2 mT, 50 Hz	Rat, male, 12 weeks old	Acute femur fracture	6 h/day 30 days	Osteoblastic material volume: Increased at 21 and 30 days (*p* < 0.05), confirmed by histomorphology analysis	(Atalay et al. 2015)
2019	DCES; Silver cathode, 100 mV/mm	Mouse fibroblasts		Once-2 h	Intracellular calcium: Increased (*p* <.01); Proliferation and cell cycle-related proteins: Elevated 24 h post-ES treatment (*p* <.001)	(Li et al. 2020)
2019	DCES; Titanium cathode, 0.3 V or 1 V, 1 Hz to 10 MHz	Human osteoblasts		20 min to 2 h/day,3 days	0.3 V, 2 h/day for 3 days: Increased ALP/total protein ratio (*p* <.05)	(Portan et al. 2019)
2020	CC; Input: 2 mT, 100 V AC Output: 60 Hz, 6 V	Rat chondrocytes		1,3 or 5 h, 4 times/day 8 days	Cell proliferation: 0.1 Vrms: Increased metabolic rate (*p* =.002), TIMP1 (*p* =.017), OPG mRNA (*p* =.005); reduced procollagen type 1 propeptide (*p* =.048)	(Stephan et al. 2020)
2020	DCES; 1 mT, 5 mT, and 10 mT, 15 Hz	Rat 3 months Male	Femur bone wound	2 h/day, 7 days	Fracture load: Higher in 5 and 10 mT groups (*p* <.05), 1 mT group: No significant difference observed	(Liu et al. 2021)
2022	PEMF; 1 V, Pulse duration: 3.6 ms, 90 V m^−1^, 12 mA, 7.9 Hz	Human osteoblast-MG-63 cells		10 min per session, 7 days	Enhanced osteoblast adhesion and modulation of calcium ion signaling	(Staehlke et al. 2022)
2023	DC Current 10 μA	Wistar rats	Calvarial bone defect	5 min; twice/week 30, 60, or 120 days	Modulated Wnt pathways, accelerated osteogenesis, improved tissue maturation	(Helaehil et al. 2023)
2023	PEMF; 0.05–0.5 mT, 10 Hz cycle, 20 kHz pulse frequency	Human patients	Acute distal radius fractures	24 h/day 6weeks	Accelerated fracture union (76% vs. 58% at 4 weeks, *p* = 0.02), shorter cast immobilization (*p* = 0.002), improved functional outcomes (SF-12, *p* = 0.005)	(Factor et al. 2023)

Among large-animal models, invasive ES, particularly DCES, yields better radiographic, histological, and biomechanical results than non-invasive methods. This may be due to difficulty delivering effective stimulation to deeper bone, where tissue resistivity and anatomy affect efficacy (Verma et al. 2022). Further research in electrical modeling is needed to ensure CC and PEMF devices deliver stable, uniform fields at fracture sites in large animals. Defining these parameters will support optimized, clinically effective protocols for human bone regeneration (Nicksic et al. 2022a; Nicksic et al. 2022b).

### Clinical applications

Clinical trials and case studies show mixed results for ES therapies. PEMFs demonstrate effectiveness in spinal fusion and fracture healing, while DC and CC methods require further optimization of electrode placement and stimulation parameters to improve outcomes (Nicksic et al. 2022a; Verma et al. 2022). High-quality level-I trials support PEMF use in various orthopedic conditions, including osteotomies, high-risk acute fractures, non-unions, stress fractures, and osteonecrosis (Vicenti et al. 2018). In fracture healing, Fontanesi et al. reported reduced healing time in acute tibial fractures treated with PEMFs, and Faldini et al. observed improved healing in femoral neck fractures following cancellous screw fixation (Vicenti et al. 2018; Faldini 2018). For osteonecrosis, PEMFs have shown benefits as a conservative therapy in early-stage hip osteonecrosis and as an adjunct to core decompression and bone grafting (Massari et al. 2019).

Massari et al. found that 53% of patients reported complete pain relief and 26% had significant pain reduction after PEMF therapy. Similarly, Santori et al. reported successful healing rates of 81% and 70% in Steinberg stage II and III hip osteonecrosis, respectively (Vicenti et al. 2018; Massari et al. 2019). For non-union fractures, PEMFs combined with mechanical stabilization achieved successful healing rates of 73–85%, depending on fracture type, patient factors, and treatment adherence (Massari et al. 2019). Cost–benefit analyses suggest early PEMF intervention may prevent non-unions, emphasizing the need to identify high-risk fractures early (Vicenti et al. 2018; Cadossi et al. 2020; Massari et al. 2018).

Recent advances in PEMF research have broadened its therapeutic applications for musculoskeletal disorders. Ongoing studies are evaluating its efficacy in bone loss after forearm fractures, acute distal radius fractures, non-union of fifth metatarsal fractures, odontoid fractures, and the preservation of bone and muscle mass in ACL reconstruction patients (clinicaltrials.gov: NCT00067834, NCT04287257, NCT00586170, NCT02281994, NCT03165318) (Vicenti et al. 2018).

**Fig. 5:**
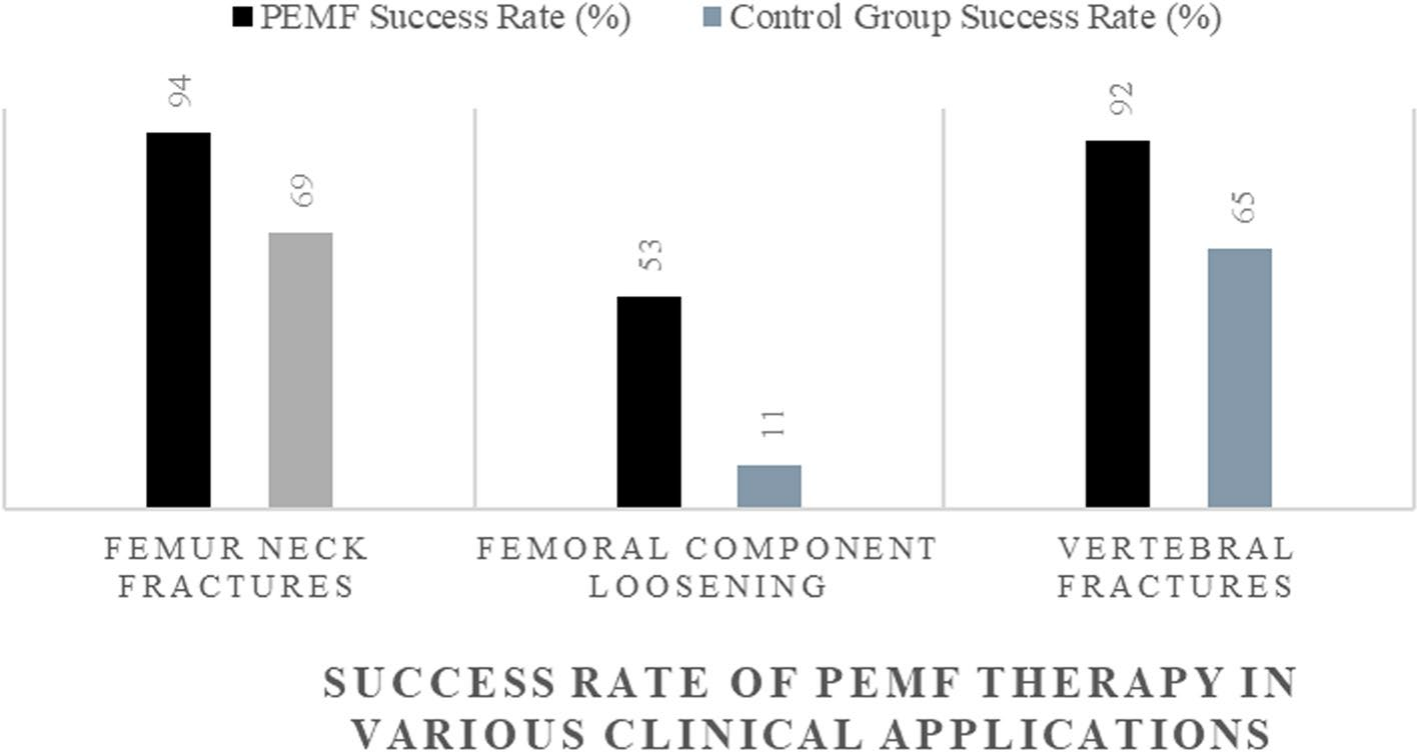
Successful healing rate of PEMF therapy in various clinical applications. The bar chart compares PEMF therapy with control groups in femoral neck fractures (94% vs. 69%), femoral component loosening (53% vs. 11%), and vertebral fractures (92% vs. 65%). Data extracted from (Faldini 2018; Kennedy 1993; Mooney 1976)

Studies confirm PEMF effectiveness in orthopedic applications. In femoral neck fractures, PEMF therapy achieved a 94% successful healing rate versus 69% in controls (Faldini 2018). In femoral component loosening, successful healing rates were 53% compared to 11% in controls (Kennedy 1993). For vertebral fractures, PEMF achieved 92% successful healing versus 65% in controls (Mooney 1976). These findings underscore PEMF’s potential as an adjunct therapy to improve bone healing and fusion outcomes (Fig. 5).

## Current FDA-approved devices and regulatory considerations

The FDA has approved various electrical bone growth stimulation devices, including PEMF and CC stimulators, for clinical use in spinal fusion, delayed fracture healing, and nonunion cases (Verma et al. 2022; Nicksic et al. 2022b; Khalifeh et al. 2018). PEMF has shown effectiveness in stimulating osteogenesis, though outcomes vary based on stimulation duration, patient compliance, and fracture location, necessitating optimized treatment parameters. EBGS devices are categorized into DCES (implantable, e.g., Zimmer Biomet DCES OsteoGen, SpF PLUS-Mini Spinal Fusion Stimulator), CC (noninvasive, e.g., SpinalPak, OrthoPak Bone Growth Stimulator Systems), and PEMF (noninvasive, e.g., EBI Bone Healing System, Cervical-Stim, Spinal-Stim). These devices fall under FDA Product Codes LOF (noninvasive) and LOE (implantable) and require premarket approval based on safety and efficacy data. However, regulatory challenges remain for emerging bioelectronic approaches such as self-powered implants and bioelectronic scaffolds, which require long-term safety and efficacy data. Further standardization of preclinical testing protocols, larger clinical trials, and improved device integration with existing orthopedic treatments will be essential for broader clinical adoption (Khalifeh et al. 2018).

## Discussion

Despite progress in ES for bone regeneration, several challenges hinder clinical adoption. A key issue is the lack of standardized protocols. Variability in intensity, frequency, duration, and electrode placement leads to inconsistent outcomes in preclinical and clinical studies, complicating guideline development and result comparison. Systematic studies are needed to define optimal parameters and improve clinical reproducibility (Nicksic et al. 2022a; Verma et al. 2022). Although short-term results are promising, long-term clinical data remains limited. While many studies show improved regeneration, data on bone integrity, remodeling, and patient-reported outcomes over time are scarce. More longitudinal studies and randomized controlled trials (RCTs) are essential to confirm the long-term benefits of ES (Anand et al. 2023).

Translating preclinical ES findings to humans is also challenging. While ES enhances osteogenesis in rodents, its effects in larger animals and humans vary due to differences in bone structure, healing rates, vascularization, and conductivity (Verma et al. 2022; Nicksic et al. 2022b). Scaling electric field parameters across species is complex, as identical settings may not yield similar biological responses. Advanced computational modeling and optimized pre-clinical studies are needed to refine ES protocols for clinical use (Ogay et al. 2020; Gu et al. 2013).

In large-animal models, implantable electrodes require long-term biocompatibility testing to assess risks such as fibrosis, infection, and mechanical failure. Patient-specific factors—including anatomy, bone density, healing capacity, age, osteoporosis, and diabetes—also affect outcomes, making standardization difficult. Personalized ES strategies tailored to individual profiles are essential (Ogay et al. 2020; Gu et al. 2013).

Device integration and invasiveness also present hurdles. Implantable ES devices, such as DC stimulators, provide localized treatment but carry risks of infection, failure, and surgical complications. Non-invasive methods like PEMF and CC require further refinement to improve efficacy, especially in deep fractures (Nicksic et al. 2022a; Verma et al. 2022).

Clinical trials must address these variabilities to develop adaptive and personalized ES therapies. Computational modeling and AI-driven approaches can help predict patient-specific responses, simulate electric field distribution, and optimize treatment parameters (Strangis et al. 2024; Zaszczy´nska et al. 2024). Innovations like injectable electrodes (Injectrode) are also being explored to improve localized stimulation in deep fractures (Verma et al. 2022).

Advancements in biomaterials, bioelectronics, and computational tools position ES as a promising strategy in regenerative medicine. Integrating ES with scaffolds has shown potential in fracture healing, spinal fusion, and dental implants (Dixon And Gomillion 2023).

Electroactive scaffolds, such as piezoelectric materials and conductive polymers like polypyrrole and polyaniline, can generate localized electric fields in response to mechanical stress, providing continuous stimulation without external power sources (Das et al. 2020; Silva et al. 2022).

Future research should focus on developing smart, bioelectronic implants and refining ES protocols. Combining ES with biochemical and mechanical stimulus such as growth factors (e.g., BMPs, VEGF), stem cell-based therapies, and mechanical loading has shown synergistic effects, enhancing osteogenesis and vascularization (Sun et al. 2023; Ogay et al. 2020). Additionally, bioelectronic innovations, including self-powered and wireless ES devices, energy-harvesting implants, and biodegradable systems, may reduce surgical risks and improve clinical accessibility (Silva et al. 2022; Dixon And Gomillion 2023).

## Conclusion

ES has emerged as a promising strategy for enhancing bone regeneration by modulating cellular behavior, promoting osteogenesis, and accelerating vascularization. This review explored the biological mechanisms of ES, the role of VGCCs, and various ES modalities, including DCES, CC, and PEMF. While preclinical studies have demonstrated the effectiveness of ES in stimulating bone healing at the cellular and tissue levels, challenges such as the lack of treatment standardization, variability in stimulation parameters, and difficulty in delivering sufficient electrical fields to deep bone structures continue to hinder its clinical translation (Verma et al. 2022; Nicksic et al. 2022b; Javeed et al. 2021).

The integration of ES with BTE, conductive biomaterials, and piezoelectric nanocomposites represents a significant advancement in regenerative medicine (Li et al. 2023; Huang et al. 2019). Piezo-electric materials, such as BaTiO3 and electroactive polymers, have shown the ability to generate localized electrical fields, mimic the natural bone environment, and enhance bone regeneration (Das et al. 2020). Additionally, 3D-printed electroactive scaffolds provide a self-powered approach to bone regeneration, enabling continuous stimulation without the need for external power sources (Sun et al. 2023; Ogay et al. 2020). However, challenges such as mechanical stability, scaffold biodegradability, and patient-specific variability must be addressed to fully implement these approaches in clinical settings (Silva et al. 2022).

In clinical applications, PEMF is the most widely used and FDA-approved ES modality, showing efficacy in treating delayed union fractures, non-union cases, and spinal fusion (Nicksic et al. 2022a; Verma et al. 2022). However, inconsistencies in treatment response, insufficient electric field penetration, and patient compliance issues remain significant barriers to widespread adoption. Large-animal studies have suggested that DCES may offer superior outcomes compared to non-invasive approaches, though risks such as infection, surgical complications, and high costs have limited its broader use (Verma et al. 2022).

Moving forward, interdisciplinary research that combines bioelectronic medicine, AI, and advanced biomaterials could significantly improve ES-based therapies (Strangis et al. 2024; Zaszczy´nska et al. 2024). Computational modeling and machine learning could facilitate the personalization of ES parameters based on individual patient conditions (Silva et al. 2022). Additionally, the development of biodegradable wireless ES implants and injectable electrode systems could help overcome existing challenges and expand clinical applications (Dixon And Gomillion 2023).

Despite the current challenges, the potential of ES as a therapeutic strategy in orthopedic and maxillofacial regenerative medicine is increasingly promising. By standardizing treatment protocols, optimizing device design, and integrating ES with next-generation biomaterials, ES-based therapies could revolutionize bone regeneration strategies, offering more efficient, personalized, and minimally invasive solutions for complex fractures and bone defects (Anand et al. 2023; Sun et al. 2023). Further clinical trials, long-term safety studies, and regulatory advancements are essential to fully realize the potential of ES in TE and regenerative medicine (Verma et al. 2022).

## Supplementary Information

Supplementary Material 1.

## Data Availability

No datasets were generated or analysed during the current study.
