# Supplementary material for: The role of electrical stimulation in bone regeneration: mechanistic insights and therapeutic advances
Source: Bioelectron Med. 2025 Aug 8;11:18. doi: 10.1186/s42234-025-00180-x (PMC12333126; doi:10.1186/s42234-025-00180-x)
Supplement: Supplementary file 1 — Supplementary Material 1. [file 42234_2025_180_MOESM1_ESM.docx]

| Table 5. Summary of Experimental Studies on Electrical Stimulation for Bone Healing Across Different Models | | | | | | |
| --- | --- | --- | --- | --- | --- | --- |
| Year | Stimulation Type  & Parameters | Model type | Osseous Injury | Duration | Outcome | Ref |
| 1976 | **CC**  0, 500, 1,000, 1,500, 2,000, 3,000 V/cm | Rat epiphyseal plate cells |  | 10 days | 500 V/cm and higher: Stimulated epiphyseal elongation.  1500 V/cm: Greatest elongation (p < .0005) | [1] |
| 1977 | **DCES**  Stainless-steel cathode  20 µA | Dog | Tibial gap osteotomy | 28 days | Histological analysis: Indicated enhanced healing (p = 0.05). | [2] |
| 1977 | **DCES**  Stainless-steel cathode  20 µA | Dog | Tibial gap osteotomy | 58 days | Histological analysis: Enhanced fibrous tissue formation and endochondral ossification (p = 0.042).  Visual assessment: Significantly improved bone healing score (p < 0.01). | [2] |
| 1979 | **CC**  5000 V/m, 60 Hz | Rat, 21-day-old Male | Fibular osteotomy | 14 days | Histomorphologic healing grades: Lower at 21 days post-treatment (p < .01). | [3] |
| 1982 | **DCES**  Stainless steel cathode with long-leg plaster cast, 20 µA | Dog | Tibial fracture | Continuous | Healing improvement: Confirmed by histomorphology analysis. | [4] |
| 1982 | **DCES**  Stainless-steel cathode, 20 µA | Rabbit, adult | Intramedullary femur | 28 days | Bone mineral density: No significant variation.  Histological assessment: No notable changes in cortical thickness. | [5] |
| 1982 | **DCES**  Platinum, stainless-steel, titanium, chromium-cobalt, gold, and silver cathodes, 0.02 and 0.2 μA/mm² | Rabbit, Male, 2 kg, adult | Intramedullary femur | 21 days | Different cathode materials optimize osteogenesis at specific current densities. | [6] |
| 1984 | **CC**  0, 10, 100, 250, 1000 V 60 Hz, 0.37 μA/mm | Bovine chondrocytes |  | 24 h | 250 V group (1% NCBS): Increased nucleotide incorporation (p < .01).  10% NCBS: Lesser increase.  10 V peak-to-peak: Decreased nucleotide incorporation (p < .01). | [7] |
| 1984 | **DCES**  Carbon fiber cathode, 1, 5, and 20 µA | Rabbit, Male, 3.5–5 kg, adult | Intramedullary tibia | 21 days | 1 μA group: Highest new bone formation.  20 μA group: Most fibrous tissue observed. | [8] |
| 1984 | **PEMF**  15 Hz, 5 ms bursts in an asymmetrical waveform | Dog  17–25 kg  2-5 years-old | Fibula bone graft | 20 h/day* | No improvement: Observed in biomechanical testing.  No significant changes: Detected in histomorphology assessment. | [9] |
| 1985 | **PEMF**  1.1 Mt | Sheep  45 kg  adult | Tibial osteotomy | 24 hours per day 6 weeks | Histology staining: No observed improvement.  Radiographs: No significant changes detected. | [10] |
| 1986 | **DCES**  Stainless-steel cathodes, 20 µA | Porcine  1- month-old | Lumbar fusion | 0–56 days | Improved healing: Assessed by radiographic fusion score (p = .037).  Increased osteoblastic activity: Confirmed by histomorphology scoring (p < .01). | [11] |
| 1987 | **DCES**  6-hole stainless steel plate | Dog  23–32 kg adult | Autologous bone graft in femur | 56 days | Torque-to-failure: No significant difference observed | [12] |
| 1988 | **DCES**  Platinum cathode,  0.1, 1 or 10 µA | Rabbit chondrocyt3.4 ± 0.2 kg  adult Male | Intramedullary tibia | 7 days | Proteoglycan synthesis: Highest at 1 µA (p < .025).  Nucleotide incorporation: Highest at 1 µA (p < .01). | [13] |
| 1988 | **CC**  5 V, 60 Hz | Rat  3- weeks-Male | Osteoporosis | 12 days | Bone mineral density: Increased by wet weight (p < .01).  Bone resorption rate: No significant difference observed.  Osteoporosis prevention: No effect detected. | [14] |
| 1990 | **DCES**  Copper cathodes,  0.1–100 µA | Dog  18–22 kg adult | Radial osteotomy (transverse, gapless) | Continuous | Improved healing at 1, 7, and 13 µA:  Biomechanical testing: Increased rigidity (p < .001, p < .002, p < .009).  Histomorphology analysis: Enhanced bone formation (p < .021, p < .004, p < .025). | [15] |
| 1994 | **PEMF**  2.5 mV | Rat  350–400 g adult | Lumbar fusion | 12 h/day  8 weeks | Micromorphological analysis: No significant differences observed.  Osteoblastic proliferation: Increased qualitatively by week 8. | [16] |
| 1995 | **DCES**  Titanium dental implant, 7.5 µA | Rabbit  5.3 ± 0.5 kg  adult | Mandibular dental implant integration (3.75 × 7.00 mm) | 28 days | No difference in torque-to-failure or histomorphology analysis | [17] |
| 1996 | **CC**  60 kHz, 3–6.3 V, 5–10 µA | Dog 11.7 ± 2.3 kg  Adult  Male | Tibial osteotomy | 28 days | Biomechanical analysis: No improvement observed.  Histologic analysis: No significant changes detected.  Radiographic analysis: No measurable improvement. | [18] |
| 1998 | **PEMF**  15 Hz, 4.5 ms assymetric pulses, 18G | Rat osteoclasts |  | Once  18 h | 1.8 mT stimulation: Led to a 2-fold increase in bone resorption (p < .009). | [19] |
| 2000 | **DCES**  40 and 100 µA groups | Sheep | Posterior lumbar spinal fusion | 4 months | 100 µA stimulation: Increased fusion score (p = .003).  Biomechanical testing: Enhanced flexion to failure (p < .029). | [20] |
| 2001 | **DCES**  Titanium cathodes,  20 and 60 µA | Rabbit  4.3 kg  adult | Lumbar fusion | 35 days | Increased radiograph fusion grades (p < .04), higher thresholds of biomechanical failure (p < .02) in 60 µA group, no difference in 20 µA group | [21] |
| 2003 | **PEMF** – 85 μsec pulse width  25 Hz frequency | Rabbit  Adult Female | Tibial dental implant integration (2.6 × 6.0 mm) | 30 min/day,5 days/wk 42 days | Torque-to-failure: No significant difference observed. | [22] |
| 2004 | **DCES**  Titanium cathode; low current density (5.4 µA/cm2)  high current density (19.6 µA /cm^2)^ | Rhesus macaques 11.3 ± 3.6 kg  Adult  Male & Female | Anterior spinal fusion (L5-6-disc space) | 84 days | Radiographic fusion grade: Reduced fusion time (p = .0001).  CT: Higher fusion rate observed (p = .0314). | [23] |
| 2004 | **PEMF**  Burst width: 4.5 ms Peak magnetic field: 1.2 mT Frequency: 15 Hz | Human umbilical vein endothelial cells | Postoperative delayed union of long-bone fracture | 8 hours/day 14 days | Stimulates endothelial release of fibroblast growth factor 2 (FGF-2) and triggers paracrine and autocrine signaling in surrounding tissues. | [24] |
| 2008 | **CC**  3–6 V, 5–10 mA,15–20 hours per day, 60 kHz | Human patients (clinical study) | Tibial stress fractures | 24 h/day  12 weeks | Increased activated calmodulin levels. | [25] |
| 2009 | **PEMF**  0.13 mT, 7.5 Hz, Efield: 2 mV/cm, 300 µs quasi-rectangular pulses | Human MSCs |  | 2 h/day  10 days | Day 7: Control had 84% more cells (p < .05); ALP increased by 82% in PEMF (p < .01).  Day 10: PEMF group had 62% more cells (p < .05); Control showed 123% cell increase. | [26] |
| 2010 | DCES Stainless-steel cathode, 50 Hz, 20 µA | Rat  Male  12-week-old | Tibial osteotomy | 20 min/day  3 weeks | Increased bone formation by histology (p < .05), callus formation measured radiograph (p < .05), increased maximal load by biomechanical testing (p < .05) | [27] |
| 2010 | **CC**  1.5 Mhz | Rat  8 weeks old Female | Osteoporosis | 20 min, 3 times/week 4 weeks | Maximal load and densitometry: No significant difference observed.  Cortical thickness: Improved, confirmed by morphometric analysis (p < .05). | [28] |
| 2010 | **CC**  10 V, 16 Hz | Rat  230–250 g Adult Female | Osteoporosis | 2 h/day  60 days | Bone mineral content: Increased, confirmed by X-ray diffraction (p < .01).  Bone mineral density: Significantly higher (p < .001). | [29] |
| 2011 | **PEMF**  1.5 mT, 2.5 mV, 75 Hz, pulse duration of 1.3 ms | Mice osteoblasts |  | Once-9 h | Day 7: Stimulated cells had 36% higher viability, 23% more total DNA, 40% less ALP activity (p < .05).  COL1: Increased.  Osteocalcin: No change. | [29] |
| 2012 | **PEMF**  0.1 mT 15 Hz and variance with 5 min cycles/ 150 Hz | Rat 220 g  20 weeks old | Osteoporosis | 2–4 h/day  5 days/week  6 weeks | No significant difference in bone density was detected using microCT scanning. | [30] |
| 2012 | **PEMF**  0.5 mT, 50 Hz | Human Bone marrow stromal cells |  | 8 h/day  12 days | Day 10: Increased  cell proliferation (p = .0312), ALP (p = .01), and  COL1 gene mRNA (p = .0001). | [31] |
| 2013 | **PEMF**  0.5 mT, 50 Hz | Human clinical research |  | 8 h/day  180 days | Higher fracture union rate (77.4%) vs. control (48.1%) (p = .029)  Reduced healing time. | [32] |
| 2013 | **PEMF**  0.4 mT 14.9 Hz | Human osteoblasts |  | 3, 7 or 10 days | Cell proliferation: Increased by 1.8%, 29%, and 55.5% on days 3, 7, and 10.  Day 10: ALP activity tripled in stimulated cells. | [33] |
| 2014 | **PEMF**  1500 μA, 12.5 Hz | Sheep  62–70 kg  2 years old | Tibial osteotomy | 12 h/a day | Callus maturation: Increased, confirmed by histology (p < .0001).  Radiodensity analysis: Active stimulation group showed increased opacity (p < .0043). | [34] |
| 2014 | **PEMF**  Screw: 0.514–0.796 V, Scaffold: 0.38–0.43 V, 20 Hz, 3 mT. | Human osteoblasts |  | 45 min/day, 3 times/day 3 days | COL1 synthesis: Increased 3-fold (p < .05).  Metabolism: Reduced (p = .026). | [35] |
| 2015 | **PEMF**  1.5 ± 0.2 mT, 50 Hz | Rat  12 weeks old Male | Acute femur fracture | 6 h/day  30 days | Osteoblastic material volume: Increased at 21 and 30 days (p < 0.05), confirmed by histomorphology analysis. | [36] |
| 2016 | **DCES**  Titanium cathode, 0.2 and 1.4 VRMS, 20 Hz | Human osteoblasts |  | 45 min,  3 times/day  3 days | 1.4 Vrms: Increased OC transcription (p = .0148).  0.2 Vrms: Increased procollagen type 1 (p = .0051). | [37] |
| 2018 | **DCES**  Stainless steel cathode, 100 V/m | Rat  9-week-old Male | Femur gap osteotomy | 1 h/day  3 weeks | ALP expression: Increased at days 7 and 14 (p < .05).  TGF-β: Elevated day 7 (p < .05).  Osteopontin and Calmodulin: Increased at all time points (p < .05). | [38] |
| 2019 | **DCES**  Lactide polymer cathode, 75 V/m | Rat bone  mesenchymal stromal cells |  | 3 h/day  21 days | ALP expression: Increased (p < .05).  COL-1 mRNA: Elevated (p < .05).  Calcium deposition: Enhanced (p < .05). | [39] |
| 2019 | **DCES**  Silver cathode, 100 mV/mm | Mouse fibroblasts |  | Once-2 h | Intracellular calcium: Increased (p < .01).  Proliferation and cell cycle-related proteins: Elevated 24 hours post-ES treatment (p < .001). | [40] |
| 2019 | **DCES**  Titanium cathode, 0.3 V or 1 V, 1 Hz to 10 MHz | Human osteoblasts |  | 20 min to 2 h/day  3 days | 0.3 V, 2 h/day for 3 days: Increased ALP/total protein ratio (p < .05). | [41] |
| 2019 | **DCES**  Platinum cathode, 2.2 V, 1 V/cm, 0.07 ± 0.01 mA | Mouse macrophages and preosteoblasts |  | 1–2 h/day  3 days | ES: Reduced both osteoclastic and osteogenic activity. | [42] |
| 2020 | **CC**  Input: 2 mT, 100 V AC Output: 60 Hz, 6 V | Rat chondrocytes |  | 1,3 or 5 h, 4 times /day  8 days | Cell proliferation: 0.1 Vrms: Increased metabolic rate (p = .002), TIMP1 (p = .017), OPG mRNA (p = .005); reduced procollagen type 1 propeptide (p = .048). | [43] |
| 2020 | **DCES**  1 mT, 5 mT, and 10 mT, 15 Hz | Rat  3 months Male | Femur bone wound | 2 h/day  7 days | Fracture load: Higher in 5 and 10 mT groups (p < .05).  1 mT group: No significant difference observed. | [44] |
| 2021 | **DCES**  0.41 mT, 1.2 mT, 4.1 mT, and 12.1 mT* | Rat  6 months Female | Osteoporosis | 3 h/day,  7 days/week | Bone mineral density: No significant difference detected using microCT scanning. | [45] |
| 2022 | **PEMF**  1V, Pulse duration: 3.6 ms  90 V m^−1^, 12 mA, 7.9 Hz | Human osteoblast-MG-63 cells |  | 10 minutes per session  7 days | Enhanced osteoblast adhesion and modulation of calcium ion signaling | [46] |
| 2023 | **DC Current**  10 μA | Wistar rats | Calvarial bone defect | 5 min  twice /week 30, 60, or 120 days | Modulated Wnt pathways, accelerated osteogenesis, improved tissue maturation | [47] |
| 2023 | **PEMF**  0.05–0.5 mT, 10 Hz cycle, 20 kHz pulse frequency | Human patients (clinical study) | Acute distal radius fractures | 24 h/day  6weeks | Accelerated fracture union (76% vs. 58% at 4 weeks, p = 0.02), shorter cast immobilization (p = 0.002), improved functional outcomes (SF-12, p = 0.005) | [48] |

[1] Zucchini, P., et al.: In vivo effects of low-frequency low energy pulsing electro- magnetic fields (pemfs) on gene expression during the inflammation phase of bone repair. Electromagn Biol Med 21(3), 197–208 (2002) [https://doi.org/10. 1081/JBC-120015991](https://doi.org/10.%201081/JBC-120015991)

[2] Paterson, D.C., Carter, R.F., Maxwell, G.M., Hillier, T.M., Ludbrook, J., Sav- age, J.P.: Electrical bone-growth stimulation in an experimental model of delayed union. The Lancet 309(8025), 1278–1281 (1977)

[3] Marino, A., Cullen, J., Reichmanis, M., Becker, R.: Fracture healing in rats exposed to extremely low-frequency electric fields. Clinical Orthopaedics and Related Research (1976-2007) 145, 239–244 (1979)

[4] Srivastava, K.P., Orth, D., Lahiri, V., Khare, A., Chandra, H.: Histomorphologic evidence of fracture healing after direct electrical stimulation in dogs. Journal of Trauma and Acute Care Surgery 22(9), 785–786 (1982)

[5] Petersson, C.J., Holmer, N.G., Johnell, O.: Electrical stimulation of osteogenesis: Studies of the cathode effect on rabbit femur. Acta Orthopaedica 53(5), 727–732 (1982) <https://doi.org/10.3109/17453678208992284>

[6] Spadaro, J.A.: Electrically enhanced osteogenesis at various metal cathodes. Journal of Biomedical Materials Research (1982)

[7] CT, U.A.S.J.B.: In vitro growth of bovine articular cartilage chondrocytes in various capacitively coupled electrical fields. Journal of Orthopaedic Research 2(1), 15–22 (1984)

[8] M, P.J.A.H.W.A.Z.: The electrical stimulation of bone using a filamentous carbon cathode. Journal of Biomedical Materials Research (1984)

[9] Miller, B.G.J., Burchardt, H., Enneking, W.F., Tylkowski, C.M.: Electromag- netic stimulation of canine bone grafts. Clinical Orthopaedics and Related Research (1984)

[10] HT, A.I.M.I.H.S.S.A.C.M.M.H.L.: The effect of induced electric currents on bone after experimental osteotomy in sheep. Journal of Bone and Joint Surgery - British Volume (1985)

[11] Nerubay, J., Marganit, B., Bubis, J.J., Tadmor, A., Katznelson, A.: Stimulation of bone formation by electrical current on spinal fusion. Spine 11(2), 167–169 (1986)

[12] Lindsey, R.W., J., G., E., L.R., M., P., E., F.G.: Effects of bone graft and electrical stimulation on the strength of healing bony defects in dogs. Clinical Orthopaedics and Related Research 222, 275–280 (1987)

[13] Okihana, H., Y., S.: Effect of direct current on cultured growth cartilage cells in vitro. Journal of Orthopaedic Research 6(5), 690–694 (1988)

[14] CT, T.G.G.S.P.S.R.B.: Treatment of denervation/disuse osteoporosis in the rat with a capacitively coupled electrical signal: effects on bone formation and bone resorption. Journal of Orthopaedic Research (1988)

[15] Chakkalakal, D.A., Lippiello, L., Shindell, R.L., Connolly, J.F.: Electrophysiology of direct current stimulation of fracture healing in canine radius. IEEE Transactions on Biomedical Engineering 37(11), 1048–1058 (1990)

[16] Guizzardi, S., Di Silvestre, M., Govoni, P., Scandroglio, R.: Pulsed electromagnetic field stimulation on posterior spinal fusions: a histological study in rats. Clinical Spine Surgery 7(1), 36–40 (1994)

[17] Shafer, D.M., Bennett, J., et al.: The effect of electrical perturbation on osseointegration of titanium dental implants: a preliminary study. Journal of Oral and Maxillofacial Surgery 53(9), (1995)

[18] Pepper, J.R., Herbert, M.A., Anderson, J.R., Bobechko, W.P.: Effect of capacitive coupled electrical stimulation on regenerate bone. Journal of Orthopaedic Research 14(2), 296–302 (1996)

[19] Shankar, V.S., Simon, B.J., Bax, C.M., Pazianas, M., Moonga, B.S., Ade- banjo, O.A., Zaidi, M.: Effects of electromagnetic stimulation on the functional responsiveness of isolated rat osteoclasts. Journal of Cellular Physiology 176(3), 537–544 (1998). https://doi.org/10.1002/(SICI)1097-4652(199803)176: 3⟨537::AID-JCP25⟩3.0.CO;2-Q

[20] Toth, J.M., Seim III, H.B., Schwardt, J.D., Humphrey, W.B., Wallskog, J.A., Turner, A.S.: Direct current electrical stimulation increases the fusion rate of spinal fusion cages. Spine 25(20), 2580–2587 (2000)

[21] France, J.C., Norman, T.L., Santrock, R.D., McGrath, B., Simon, B.J.: The efficacy of direct current stimulation for lumbar intertransverse process fusions in an animal model. Spine 26(9), 1002–1007 (2001)

[22] Buzz´a, E.P., Shibli, J.A., Barbeiro, R.H., A. Barbosa, J.R.: Effects of electromagnetic field on bone healing around commercially pure titanium surface: Histologic and mechanical study in rabbits. Implant Dentistry 12(2), 182–187 (2003) <https://doi.org/10.1097/01.ID.0000058385.23346.4D>

[23] Cook, S.D., Patron, L.P., Christakis, P.M., Bailey, K.J., Banta, C., Glazer, P.A.: Direct current stimulation of titanium interbody fusion devices in primates. The Spine Journal 4(3), 300–311 (2004) [https://doi.org/10.1016/j.spinee.2003. 12.005](https://doi.org/10.1016/j.spinee.2003.%2012.005)

[24] Tepper, O.M., Callaghan, M.J., Chang, E.I., Galiano, R.D., Bhatt, K.A., Baharestani, S., Gan, J., Simon, B., Hopper, R.A., Levine, J.P., Gurtner, G.C.: Electromagnetic fields increase in vitro and in vivo angiogenesis through endothelial release of fgf-2. The FASEB Journal 18(11), 1231–1233 (2004). <https://doi.org/10.1096/fj.03-0967fjeb>

[25] Beck, B.R., et al.: Do capacitively coupled electric fields accelerate tibial stress fracture healing?: A randomized controlled trial. American Journal of Sports Medicine 36(3), 545–553 (2008) <https://doi.org/10.1177/0363546507310076>

[26] Tsai, M.T., Li, W.J., Tuan, R.S., Chang, W.H.: Modulation of osteogenesis in human mesenchymal stem cells by specific pulsed electromagnetic field stimulation. Journal of Orthopaedic Research 27(9), 1169–1174 (2009). https: //doi.org/10.1002/jor.20862

[27] Nakajima, M., et al.: Effect of electroacupuncture on the healing process of tibia fracture in a rat model: A randomised controlled trial. Acupuncture in Medicine 28(3), 140–143 (2010) <https://doi.org/10.1136/aim.2009.001800>

[28] Manjhi, J., et al.: Effect of low level capacitive coupled pulsed electric field stimulation on mineral profile of weight-bearing bones in ovariectomized rats. Journal of Biomedical Materials Research 92(1), 189–195 (2010)

[29] Lin, H.-Y., Lin, L.Y.-J.: In vitro effects of low frequency electromagnetic fields on osteoblast proliferation and maturation in an inflammatory environment. Bioelectromagnetics 32(7), 552–560 (2011)

[30] Van Der Jagt, O.P., Van Der Linden, J.C., Waarsing, J.H., Verhaar, J.A.N., Weinans, H.: Systemic treatment with pulsed electromagnetic fields do not affect bone microarchitecture in osteoporotic rats. International Orthopaedics 36(7), 1501–1506 (2012) <https://doi.org/10.1007/s00264-011-1471-8>

[31] Zhong, H.R., et al.: Effects of low-intensity electromagnetic fields on the proliferation and differentiation of cultured mouse bone marrow stromal cells. Physical Therapy 92(9), 1208–1219 (2012)

[32] Shi, H.F., et al.: Early application of pulsed electromagnetic field in the treatment of postoperative delayed union of long-bone fractures: A prospective randomized controlled study. BMC Musculoskeletal Disorders 14, 35 (2013) <https://doi.org/10.1186/1471-2474-14-35>

[33] Barnaba, S., Papalia, R., Ruzzini, L., Sgambato, A., Maffulli, N., Denaro, V.: Effect of pulsed electromagnetic fields on human osteoblast cultures. Physiotherapy Research International 18(2), 109–114 (2013). https://doi.org/10.1002/pri. 1536

[34] Muttini, A., et al.: Effect of electric current stimulation in combination with external fixator on bone healing in a sheep fracture model. Veterinaria Italiana 50(4), 249–257 (2014) <https://doi.org/10.12834/VetIt.271.963.2>

[35] Grunert, P.C., et al.: Establishment of a novel in vitro test setup for electric and magnetic stimulation of human osteoblasts. Cell Biochemistry and Biophysics 70(2), 805–817 (2014)

[36] Atalay, Y., Gunes, N., Guner, M.D., Akpolat, V., Celik, M.S., Guner, R.: Pen- toxifylline and electromagnetic field improved bone fracture healing in rats. Drug Design, Development and Therapy 9, 5195–5201 (2015) https://doi.org/ 10.2147/DDDT.S89669

[37] Dauben, T.J., Ziebart, J., Bender, T., Zaatreh, S., Kreikemeyer, B., Bader, R.: A novel in vitro system for comparative analyses of bone cells and bacteria under electrical stimulation. Biomed Research International 2016 (2016). https: //doi.org/10.1155/2016/5178640

[38] Leppik, L., et al.: Combining electrical stimulation and tissue engineering to treat large bone defects in a rat model. Scientific Reports 8(1) (2018) https: //doi.org/10.1038/s41598-018-24892-0

[39] Jing, W., et al.: Roles of electrical stimulation in promoting osteogenic differentiation of bmscs on conductive fibers. Journal of Biomedical Materials Research A 107(7), 1443–1454 (2019)

[40] Li, Y., et al.: A novel pulsed electromagnetic field promotes distraction osteo- genesis via enhancing osteogenesis and angiogenesis in a rat model. Journal of Orthopaedic Translation 25, 87–95 (2020) [https://doi.org/10.1016/j.jot.2020. 10.007](https://doi.org/10.1016/j.jot.2020.%2010.007)

[41] Portan, D.V., Deligianni, D.D., Papanicolaou, G.C., Kostopoulos, V., Psarras, G.C., Tyllianakis, M.: Combined optimized effect of a highly self-organized nanosubstrate and an electric field on osteoblast bone cells activity. Biomed Research International 2019 (2019) <https://doi.org/10.1155/2019/7574635>

[42] Srirussamee, K., Mobini, S., Cassidy, N.J., Cartmell, S.H.: Direct electrical stimulation enhances osteogenesis by inducing bmp2 and spp1 expressions from macrophages and preosteoblasts. Biotechnology and Bioengineering 116(12), 3421–3432 (2019) <https://doi.org/10.1002/bit.27142>

[43] Stephan, M., et al.: Establishment and evaluation of an in vitro system for biophysical stimulation of human osteoblasts. Cells 9(9), 1–19 (2020) https:// doi.org/10.3390/cells9091995

[44] Liu, Y., et al.: Therapeutic effect of pulsed electromagnetic field on bone wound healing in rats. Electromagnetic Biology and Medicine 40(1), 26–32 (2021)

[45] Androjna, C., et al.: A comparison of alendronate to varying magnitude pemf in mitigating bone loss and altering bone remodeling in skeletally mature osteoporotic rats. Bone 143, 115761 (2021)

[46] Staehlke, S., et al.: Pulsed electrical stimulation affects osteoblast adhesion and calcium ion signaling. Cells 11(17) (2022) https://doi.org/10.3390/ cells11172650

[47] Helaehil, J.V., et al.: Electrical stimulation therapy and ha/tcp composite scaffolds modulate the wnt pathways in bone regeneration of critical-sized defects. Bioengineering 10(1) (2023) <https://doi.org/10.3390/bioengineering10010075>

[48] Factor, S., et al.: The effects of novel pulsed electromagnetic field therapy device on acute distal radius fractures: A prospective, double-blind, sham-controlled, randomized pilot study. J Clin Med 12(5) (2023) https://doi.org/10.3390/ jcm12051866
